# The effect of pregabalin or duloxetine on arthritis pain: a clinical and mechanistic study in people with hand osteoarthritis

**DOI:** 10.2147/JPR.S147640

**Published:** 2017-10-10

**Authors:** Nidhi Sofat, Abiola Harrison, Mark D Russell, Salma Ayis, Patrick D Kiely, Emma H Baker, Thomas Richard Barrick, Franklyn A Howe

**Affiliations:** 1 Institute for Infection and Immunity, St George’s University of London; 2 Division of Health & Social Care Research, King’s Clinical Trials Unit, King’s College London; 3 Department of Rheumatology, St George’s University Hospitals NHS Foundation Trust; 4 Molecular and Clinical Sciences Research Institute, St George’s University of London, London, UK

**Keywords:** pain, hand osteoarthritis, sensitization, duloxetine, pregabalin

## Abstract

Osteoarthritis (OA) is the most prevalent arthritis worldwide and is characterized by chronic pain and impaired physical function. We hypothesized that heightened pain in hand OA could be reduced with duloxetine or pregabalin. In this prospective, randomized clinical study, we recruited 65 participants, aged 40–75 years, with a Numerical Rating Scale (NRS) for pain of at least 5. Participants were randomized to one of the following three groups: duloxetine, pregabalin, and placebo. The primary endpoint was the NRS pain score, and the secondary endpoints included the Australian and Canadian Hand Osteoarthritis Index (AUSCAN) pain, stiffness, and function scores and quantitative sensory testing by pain pressure algometry. After 13 weeks, compared to placebo, ANOVA found significant differences between the three groups (*P*=0.0078). In the intention-to-treat analysis, the pregabalin group showed improvement for NRS pain (*P*=0.023), AUSCAN pain (*P*=0.008), and AUSCAN function (*P*=0.009), but no difference between duloxetine and placebo (*P*>0.05) was observed. In the per protocol analysis, NRS pain was reduced for pregabalin (*P*<0.0001) and duloxetine (*P*=0.029) compared to placebo. We conclude that centrally acting analgesics improve pain outcomes in people with hand arthritis, offering new treatment paradigms for OA pain.

## Introduction

Osteoarthritis (OA) is the most common form of arthritis worldwide, with hand pain and reduced function causing significant problems for people with hand OA.^1^ Pain is a major symptom for people with OA, with 16.7% of US adults aged 45 years and older reporting pain as a predominant problem.^2^ Pain and reduced function due to OA place a huge burden on patients and health care services.^2^,^3^ Although several pharmacological agents are available for OA pain management, including acetaminophen, nonsteroidal anti-inflammatory drugs (NSAIDs), and opioids, a large proportion of patients continue to suffer from chronic pain despite using the agents described.^4^,^5^ Recent trials have raised questions about current treatments, suggesting that acetaminophen has poor efficacy in controlling OA pain.^6^,^7^ Pain management in OA is a huge problem, and novel approaches are urgently needed.

Pain is often characterized as having features of inflammatory nociceptive pain and neuropathic components.^8^,^9^ OA is recognized to have features of inflammatory pain and also pain sensitization. Features of pain sensitization can be evaluated using quantitative sensory testing (QST)^10^–^13^ and brain neuroimaging.^14^–^16^ Large studies have shown pain sensitization using QST in knee OA.^10^–^13^ Brain neuroimaging studies in chronic OA have also demonstrated increased central pain processing in the cingulate cortex, insula, and thalamus compared with normal controls.^14^,^15^ Many clinical trials testing new agents for OA have focused on large joint hip and knee OA, but relatively few trials have been conducted in hand OA. The ideal analgesic drug(s) in OA would achieve sustained pain relief in a dose-dependent manner with few side effects. Centrally acting analgesic drugs such as pregabalin and duloxetine could fulfill these criteria but have not been investigated in hand OA. Duloxetine is a serotonin and noradrenaline reuptake inhibitor (SNRI) that has shown efficacy for improving pain in knee OA.^17^ However, no previous studies have evaluated duloxetine in hand OA. Gabapentinoids are three-substituted derivatives of the neurotransmitter gamma-aminobutyric acid that blocks voltage-dependent calcium channels, used to treat epilepsy and neuropathic pain. Interest has grown in gabapentinoids for arthritis, since gabapentin inhibits pain sensitization.^18^,^19^ Arthritic pain can be improved by NSAIDs and pregabalin in OA.^20^,^21^ Ohtori et al^20^ found that pregabalin combined with meloxicam was more effective for knee OA pain compared to either drug alone and Arendt-Nielsen et al^21^ showed that pain sensitization is improved by NSAIDs in knee OA.

We hypothesized that centrally acting analgesics may alleviate arthritic pain. We conducted a proof-of-concept, randomized, placebo-controlled study comparing duloxetine and pregabalin to placebo for hand OA pain. We used validated primary endpoints for pain, with secondary endpoints for pain sensitization using QST, depression, and anxiety scores. Our report is the first proof-of-concept clinical trial comparing the effect of centrally acting analgesics duloxetine and pregabalin head-to-head vs placebo in hand OA pain with mechanistic secondary endpoints for pain threshold testing.

## Methods

### Study design and participants

All methods were carried out in accordance with relevant guidelines and regulations. All trial protocols were approved by the sponsors, St George’s University of London and the Medicines and Healthcare Products Regulatory Agency, UK. Ethical approval was provided by the London-Surrey Borders Ethics Committee, approval number 12/LO/0047. Written informed consent was obtained from all subjects. The clinical trial registration number is NCT02612233. Participants were eligible if they were aged 40–75 years and had hand OA diagnosed by American College of Rheumatology (ACR) criteria^22^,^23^ confirmed by a rheumatologist and experiencing pain of at least ≥5 on a Numerical Rating Scale (NRS) of 0–10. The trial protocol was followed as published, according to CONSORT guidelines and inclusion–exclusion criteria. Twenty age-matched subjects without hand OA were enrolled as controls for comparisons for pain testing and brain MRI. The brain MRI data from this study will be reported in a separate publication.

### Inclusion and exclusion criteria

Inclusion criteria were as follows: participants fulfilling the ACR criteria for the diagnosis of hand osteoarthritis, male or female, right or left handed, aged 40–75 years, and on usual care for hand OA including acetaminophen and/or NSAIDs. Exclusion criteria were another rheumatological diagnosis, eg, rheumatoid arthritis, current or planned pregnancy, contraindications to duloxetine or pregabalin such as concomitant use of monoamine oxidase inhibitors, selective serotonin reuptake inhibitors, antidepressants, oral contraceptives, St. John’s wort, history of depression, concomitant use of opioids including tramadol and pethidine, use of benzodiazepines, recent surgery, ie, <6 weeks prior to participation in the study, recent insertion of surgical implants, ie, <6 weeks before participation prior to entry, previous use of duloxetine and/or pregabalin, uncontrolled depression, estimated glomerular filtration rate <60 mL/min, hepatic impairment defined as ALT >2.5× upper limit of normal within 6 weeks of last clinical assessment, ischemic heart disease, diabetes mellitus, and regular use of alcohol or alcohol abuse (maximum limits are 28 units/week for men and 21 units/week for women, lactose intolerance). The estimated glomerular filtration rate was checked by screening blood tests, and any participants who were outside the stated range were not enrolled. Uncontrolled hypertension was checked by blood pressure in primary care, and any participants with a blood pressure >140/90 were excluded. Baseline laboratory tests of renal function and hepatic function were performed at baseline to screen for any impairment, and participants with levels outside the normal range were excluded. We checked information from all participants about a new diagnosis of diabetes, and any new cases were excluded. For the Hospital Anxiety and Depression Scale (HADS) scoring, a score of ≥12 for anxiety and/or depression was considered as too high for enrollment to the study and participants with a score of >12 were excluded.

### Randomization and masking

Study drugs were supplied by Sharp Clinical Services (formerly Bilcare GCS, Powys, UK), which overencapsulated pregabalin 150 mg tablets or duloxetine 30 mg tablets and produced visually identical placebo capsules. A mid-ranging dose was selected for each of the trial medications. The random allocation sequence, with a block size of nine, was generated by the manufacturer and implemented through sequentially numbered containers. Neither participants nor investigators were aware of treatment assignment until after completion of the trial, which was performed after the last patient and last visit were conducted at the end of the trial. Emergency code breaks were administered independently by the staff from the St George’s University Hospitals NHS Foundation Trust’s Clinical Trials Pharmacy.

### Clinical outcome measures

The primary endpoints were the NRS and the Australian and Canadian Hand Osteoarthritis Index (AUSCAN) rating scale 3.1 for pain,^24^,^25^ which are validated outcome measures for pain. Both NRS and AUSCAN pain endpoints are well-recognized primary endpoints in hand OA clinical trials and have been recommended in international guidelines.^25^,^28^

Prespecified secondary endpoints included the AUSCAN stiffness and function scales and HADS^26^ at baseline and after 12 weeks treatment. All endpoints were specified prospectively.

### Pain algometry

Pain pressure thresholds (PPTs) were used to obtain objective measures of peripheral pain sensitization as we described previously.^27^ Briefly, a calibrated digital hand held algometer (FDX 100; Wagner Instruments, Greenwich, CT, USA) was used for all measurements. A standard operating procedure was used, which consisted of testing pain thresholds in all participants in both hands with n=30 regions for each participant, 780 regions in total. Regions tested included dorsal aspects of all distal interphalangeal, proximal interphalangeal and metacarpophalangeal joints of each digit and thumb and the dorsum of each wrist. The 1 cm^2^ flat rubber algometer probe was held perpendicular to the dorsal aspect of the skin, and force was applied to provide a constant increase in pressure at a rate of 1 N/cm^2^/s. Therefore, the algometer scores are stated as Newton per centimeter squared in all reported results. The individual was asked to say “stop” when the sensation of pressure became the first sensation of pain. The algometer was applied to each joint being examined three times in succession with an interval between applications. After all three readings were taken, the average from the last two readings was calculated as the PPT. The intervals between each algometer measurement were long enough to prohibit temporal summation.

### Statistical analysis

Our sample size was based on IMMPACT guidelines^25^ and OARSI recommendations for RCTs in hand OA^28^ using NRS pain for the sample size calculation. For the NRS pain outcome, we aimed to detect a mean difference of 2.0 (SD 1.9) points between baseline and treatment after 12 weeks. With 16 participants in each group, 80% power with a 0.05 significance level (two sided) is achieved. Recruitment required up to 22 participants per treatment group, allowing a dropout rate of 25%, giving a total intervention study number of 65 participants to achieve desired statistical power.

Planned analyses included initial comparison to detect any significant differences between baseline and 13-week timepoints using primary endpoint NRS and AUSCAN pain difference between all three groups by ANOVA, with a multiple comparisons test, alpha =0.05. Following ANOVA, pairwise comparisons were performed for placebo vs pregabalin and placebo vs duloxetine. The intent-to-treat analysis was performed using the last observation at week 13 and carried forward for all participants. We present the NRS pain and AUSCAN pain, stiffness, and function outcomes as mean and confidence interval for all analyses. These are presented after checking the distribution of the data, which followed a normal distribution and were not skewed for the parameters measured. We also show the per protocol analysis for all completers.

**Figure 1:**
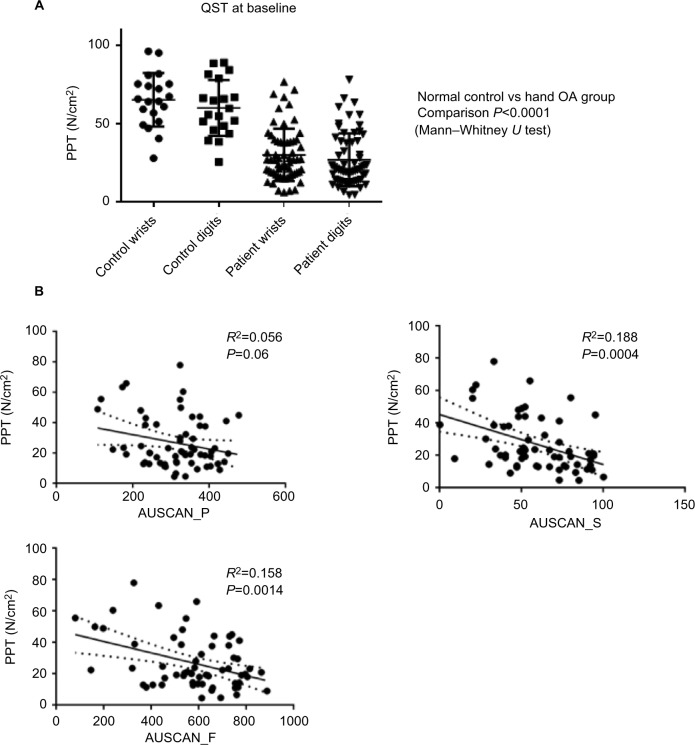
Pain sensitization characteristics of study population. **Notes:** (**A**) Data from the DUPRO clinical trial demonstrating reduced pain thresholds globally in the wrist and finger joints in hand OA participants compared to normal age- and sex-matched controls. (**B**) Graphs demonstrating correlation for PPT in Newton per centimeter squared at baseline with clinical measures for AUSCAN_P, AUSCAN_S, and AUSCAN_F in all groups. **Abbreviations:** AUSCAN_P, Australian and Canadian Hand Osteoarthritis Index pain; AUSCAN_S, Australian and Canadian Hand Osteoarthritis Index stiffness; AUSCAN_F, Australian and Canadian Hand Osteoarthritis Index function; OA, osteoarthritis; PPT, pain pressure threshold; QST, quantitative sensory testing.

For the comparison of pain pressure algometry (PPT) in non-OA vs OA participants, Mann–Whitney *U* was used (Figure 1A). For correlation analyses between AUSCAN scores and PPT, an *R*^2^ correlation and *P*-value were calculated using GraphPad Prism (Figure 1B). In the bivariate comparisons of clinical outcome measures and PPT, SPSS was used to calculate an *R*^2^ correlation and *P*-value (Table 1).

We used IBM SPSS Statistics 21.0 for all analyses. Graphs were plotted using SPSS or GraphPad Prism Version 7.

## Results

### Characteristics of patient population

**Figure 2:**
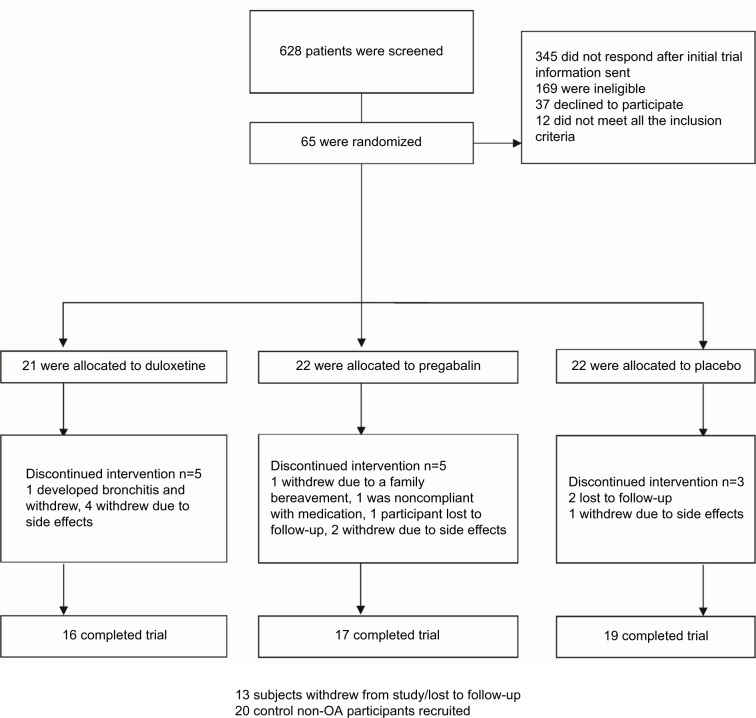
CONSORT flow diagram for the DUloxetine or PRegabalin for Osteoarthritis pain (DUPRO) clinical trial.

**Figure 3:**
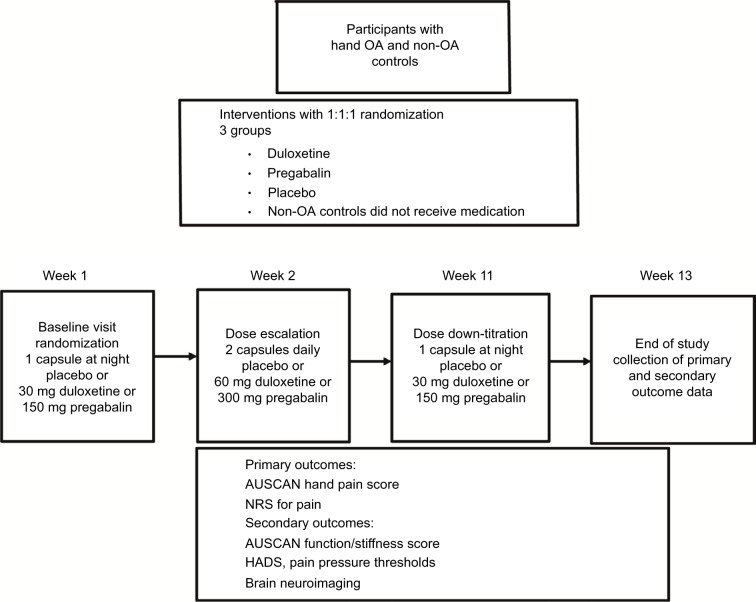
Study flow diagram and outcome measures. **Abbreviations:** AUSCAN, Australian and Canadian Hand Osteoarthritis Index; HADS, Hospital Anxiety and Depression Scale; NRS, Numerical Rating Scale; OA, osteoarthritis.

Between April 2013 and April 2016, we recruited 65 participants (Figure 2). A total of 21 participants were randomized to duloxetine, a further 22 participants were randomized to pregabalin, and 22 participants were randomized to placebo, respectively. There were 20 age-matched healthy volunteer participants enrolled for the comparison of pain scores using algometry and brain MRI (MRI data from this study will be reported separately). All 65 participants who were randomized to treatment were included in the intention-to-treat (ITT) analysis. A total of 52 participants completed the trial procedures after 13 weeks and were included in the per protocol analyses (Figure 3). Baseline characteristics show that the three treatment groups were well-matched for demographic data (Table 2). The mean disease duration was 3.5 years (SD 4.2), which was measured from the time that the participant was first told that they had a diagnosis of hand OA. For prior analgesic use, there was slightly less acetaminophen use at baseline before enrollment in the duloxetine group than in the pregabalin and placebo groups, but for other NSAIDs and opiates, analgesic use was similar in all three groups.

### Patient-reported outcomes

#### ITT analysis

Participants in all three groups receiving duloxetine, pregabalin, or placebo reported improvement in pain at the end of the trial. Comparison of the three groups by ANOVA showed a significant difference at the end of treatment for NRS pain (*P*=0.035) and AUSCAN pain (*P*=0.0078) at the end of the trial. Following the primary analysis, pairwise comparisons were performed.

### Pregabalin

**Figure 4:**
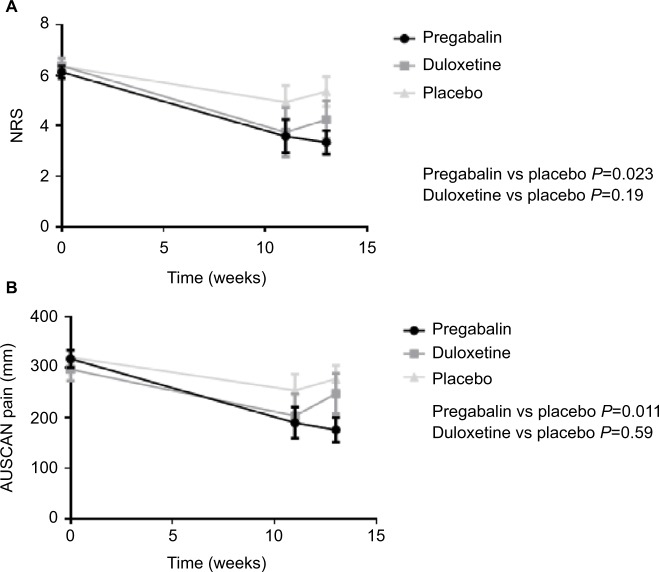
(**A, B**) Plots for change in primary outcome measures in all treatment groups (ITT analysis). **Abbreviations:** AUSCAN, Australian and Canadian Hand Osteoarthritis Index; ITT, intention-to-treat; NRS, Numerical Rating Scale.

Comparison of pregabalin vs placebo showed a significant improvement in the pregabalin group for primary outcomes of NRS pain (*P*=0.023), AUSCAN pain (*P*=0.008), and AUSCAN function (*P*=0.009) but not AUSCAN stiffness (*P*=0.22) scores (Table 3 and Figure 4).

### Duloxetine

For NRS pain and AUSCAN pain, function, and stiffness outcomes in patients receiving duloxetine compared to placebo, none of these outcomes were significantly different to placebo (Table 3).

### Use of rescue medication

Average use of acetaminophen as rescue medication was much lower in the pregabalin and duloxetine groups than in the placebo group (Table 3). The use of rescue medication in the placebo group was higher, amounting to 56 days.

#### Per protocol analysis

There was a reduction in reporting pain in all three groups at the end of the trial. A significant difference between the three groups at the end of treatment for NRS pain score (*P*=0.04) was found by ANOVA. Pairwise comparisons between duloxetine and placebo, pregabalin, and placebo were then performed (Table 4).

### Pregabalin

For NRS pain, pregabalin was more effective than placebo (*P*<0.0001). Similarly, compared to placebo, there was a significant improvement in the pregabalin group for AUS-CAN pain (*P*=0.013), AUSCAN function (*P*=0.02) but not AUSCAN stiffness (*P*=0.06).

### Duloxetine

For the comparison between placebo and duloxetine treatment, duloxetine was more effective as measured by NRS after 13 weeks (*P*=0.029). For AUSCAN pain, stiffness, and function outcomes in patients receiving duloxetine, these outcomes did not reach statistical significance.

#### Adverse events

**Figure 5:**
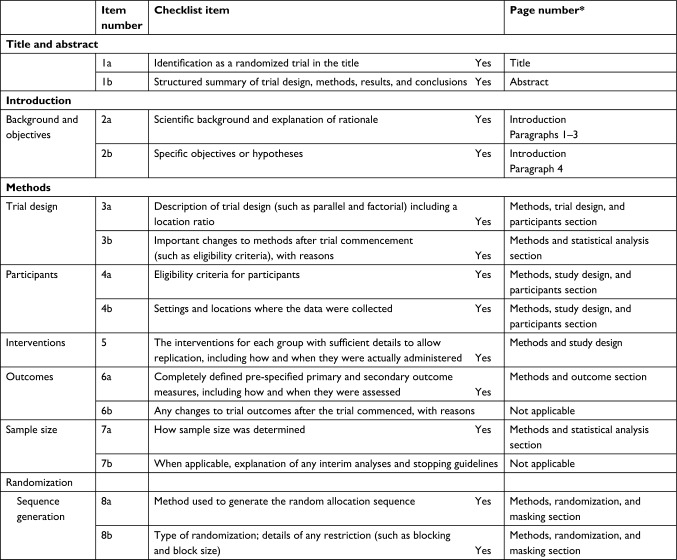

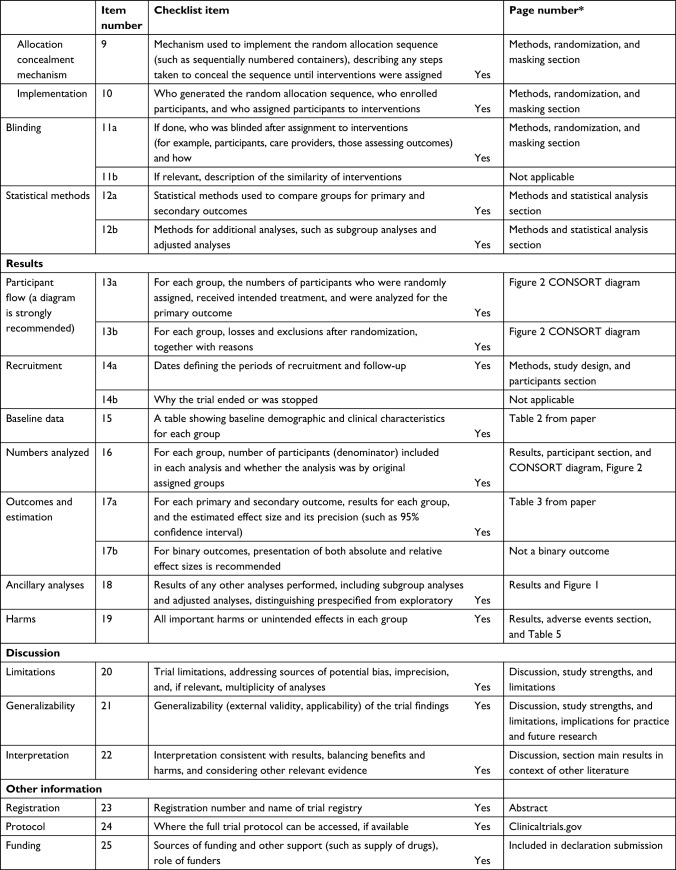
CONSORT 2010 checklist of information for the DUloxetine or PRegabalin for Osteoarthritis pain (DUPRO) randomized controlled trial. **Note:** *Page numbers optional depending on journal requirements. Hopewell S, Clarke M, Moher D, Wager E, Middleton P, Altman DG, et al. CONSORT for reporting randomised trials in journal and conference abstracts. *Lancet*. 2008;371:281–283.^38^

Side effects were recorded prospectively throughout the study (Table 5). The placebo group showed fewer adverse events with a total of 22 recorded, with no difference in adverse events between the three groups (*P*=0.73). The highest reporting of adverse events was observed in the pregabalin and duloxetine groups: 55 adverse events were recorded with pregabalin, the most common of which were mental disturbance, headaches, sleepiness, dizziness, and dry mouth. In the duloxetine group, a total of 57 adverse events were recorded; there were a total of four withdrawals due to drug side effects and one participant withdrew due to the development of bronchitis, as shown in the CONSORT flow diagram (Figures 3 and 5). For the pregabalin group, there were two withdrawals due to drug side effects, one withdrawal due to a family bereavement, one withdrawal due to noncompliance, and one withdrawal due to loss to follow-up.

#### Pain sensitization by PPT and relation to clinical scores

Using PPT testing as a measure for pain sensitization, compared to non-OA controls, the hand arthritis group had globally reduced pain thresholds (*P*<0.0001) across all finger joints at baseline, even at the metacarpophalangeal joints and wrists where there was little evidence of radiographic OA (Figure 1A). We investigated the correlation between the various clinical scores at baseline with age as a covariate (Table 1). Measurements for the PPT modality of QST showed a significant correlation with AUSCAN stiffness (*R*^2^=0.188, *P*=0.0004) and function (*R*^2^=0.158, *P*=0.0014) for all patients at baseline (Figure 1B). There was a trend for lower PPT correlated with higher AUSCAN pain scores, although this trend did not reach statistical significance (*P*=0.06). NRS pain correlated strongly with AUSCAN pain and function (*P*<0.0001). PPT measures did not change significantly in any of the three groups after 3 months treatment. We found that all participants with hand OA had lower PPT scores at baseline compared to healthy controls at inclusion and demonstrated a reduction of NRS at follow-up. HADS anxiety and depression scores were significantly correlated after Bonferroni correction. There were weaker correlations (significant without correction) between HADS depression and AUSCAN pain (*P*=0.009) and between AUSCAN function and stiffness scores (*P*=0.008).

## Discussion

### Principal findings

Our clinical study provides the first evidence in chronic painful hand OA that pregabalin and duloxetine are analgesics with potential for use in OA pain, with pregabalin providing the best treatment response and sustained effects beyond the reduction in dose. Second, we observed by QST that hand arthritis subjects have pain sensitization, which may include peripheral and central mechanisms. Third, the central but distinct actions of pregabalin and duloxetine could therefore be exerting an effect on central pain sensitization, which we and others have demonstrated as a significant component of arthritic pain.^14^,^15^ Finally, we observed improvement for pregabalin in NRS for the ITT analysis, but not for duloxetine, with improvement in NRS for both active drugs only in the per protocol analysis. The results of our trial have strong clinical relevance, since many patients report lack of efficacy or side effects on NSAIDs and other patients have important safety concerns.

### Study strengths and limitations

The lack of new analgesic targets for OA in this most common arthritic disease, coupled with recent data from animal models,^18^,^19^ prompted us to investigate the use of the gabapentinoid pregabalin and the SNRI duloxetine. Pregabalin is licensed for neuropathic pain^29^ and duloxetine for depression and diabetic neuropathic pain.^30^ Our proof-of-concept trial demonstrated an improvement in pain for pregabalin and also for duloxetine after 13 weeks treatment. We enrolled subjects who had an NRS pain rating of at least 5 to ensure that clinically meaningful improvements in pain could be detected. There were some differences in our ITT and per protocol analysis: in ITT, pregabalin, but not duloxetine, showed a significant improvement in pain compared to placebo; and in the per protocol analysis, both agents showed an improvement in pain. Although we observed an improvement in pain reporting for both centrally acting agents, pregabalin was more effective after 13 weeks. In our secondary endpoint analyses, we did not see any significant improvement in depression or anxiety scores in any treatment group.

Since this was a proof-of-concept analgesic endpoint study, we did not collect structural outcome data including joint damage progression changes by plain radiograph and synovitis by ultrasound, as described in other studies,^31^–^34^,^37^ which could be addressed in future work. We also recognize that our study has been conducted in hand arthritis pain, whereas several large previous datasets have focused on knee OA,^35^–^37^ and there may be some differences in pain characteristics including loading effects due to structural joint differences between the hand and knee.

### Main results in context of other literature

In knee OA, Chappell et al^17^ showed that duloxetine was effective for pain, Ohtori et al^20^ found that pregabalin with meloxicam was more effective than pregabalin alone, and our data show that both agents have efficacy in chronic hand OA pain with pregabalin showing superiority over duloxetine.

Recent concepts in novel therapeutic agents for OA have included potential therapeutics for structural changes in the joint including synovitis^31^ and bone marrow lesions (BML).^32^ However, such studies have not been without difficulty since recent trials targeting the inflammatory component of OA have not shown improved outcomes^33^ and the use of bisphosphonates potentially for reducing BML-related pain need to define significant clinical and structural endpoints.^34^ It is possible that patients demonstrating a largely “inflammatory” phenotype are likely to benefit from agents such as NSAIDs, and when there are features of sensitization with ongoing pain, patients may require additional treatment such as centrally acting agents including pregabalin and duloxetine. In the clinic, patients may also require additional treatments if NSAIDs are linked to side effects, lack of efficacy, and ongoing pain.

There is recognition that pain sensitization occurs in people with OA.^11^–^15^,^35^–^37^ The main indication from our data of peripheral sensitization in hand OA is that the control subjects had significantly higher PPTs than the hand OA group. We noted that PPTs did not change significantly after treatment, suggesting that pathways which led to sensitization in hand arthritis may continue to exist in the patients even after drug treatment.

### Implications for practice and future research

Pregabalin and duloxetine had efficacy in hand OA pain in our clinical study, with pregabalin showing greater effect than duloxetine for validated pain endpoints. In our study, one or more of the following analgesics had been used by more than half of the participants prior to enrollment in the study: acetaminophen, NSAID, or codeine-based analgesics. When such analgesics had not previously been effective, our trial showed that pregabalin, and to a less significant degree duloxetine, may provide a realistic alternative to pain management in OA. In future, clinical trials that examine the efficacy of centrally acting analgesics over a longer treatment period of >12 weeks in chronic arthritic pain should be conducted. Further studies measuring peripheral and central sensitization will be crucial to understand how pain, loss of function, comorbid conditions, and medication use contribute to the development of arthritic pain.

## Figures and Tables

**Table 1:** Bivariate correlation analysis of clinical scores in study

	AUSCAN_P	AUSCAN_S	AUSCAN_F	QST	HADS_A	HADS_D
NRS	0.606	0.268	0.471	−0.167	−0.010	0.158
	0.000	0.034	0.000	0.203	0.938	0.216
	AUSCAN_P	0.251	0.716	−0.234	0.171	0.328
		0.047	0.000	0.072	0.179	0.009
		AUSCAN_S	0.331	−0.429	0.230	0.213
			0.008	0.001	0.070	0.093
			AUSCAN_F	−0.409	0.132	0.294
				0.001	0.304	0.019
				QST	−0.285	−0.187
					0.027	0.152
					HADS_A	0.692
						0.000

**Abbreviations:** AUSCAN_P, Australian and Canadian Hand Osteoarthritis Index pain; AUSCAN_S, Australian and Canadian Hand Osteoarthritis Index stiffness; AUSCAN_F, Australian and Canadian Hand Osteoarthritis Index function; HADS, Hospital Anxiety and Depression Scale; NRS, Numerical Rating Scale; QST, quantitative sensory testing.

**Table 2:** Baseline characteristics of study patients (ITT analysis)

Characteristics	Pregabalin (n=22)	Duloxetine (n=21)	Placebo (n=22)
Age (years), mean (SD)	64.0 (5.0)	62.3 (7.3)	62.4 (8.7)
Women	19 (86.4)	14(66.7)	19 (86.4)
White	20 (90.9)	20 (95.2)	18 (81.8)
Black	2 (9.1)	1 (4.8)	1 (4.6)
Asian			3 (13.6)
Body mass index, mean (SD)	27.1 (6.3)	28.4 (5.9)	27.0 (4.3)
NRS, mean (SD)	6.1 (1.2)	6.4 (1.5)	6.4 (1.4)
AUSCAN pain score, mean (SD)	317.0 (81.4)	296.0 (105.2)	320.3 (66.2)
HADS, mean (SD)	11.6 (7.4)	10.3 (6.1)	12.2 (6.2)
Most common analgesics before inclusion			
Acetaminophen	15	8	15
Other NSAID oral/topical	7	5	5
Codeine-based analgesic	3	4	6

**Note:** Values are numbers (percentages) unless stated otherwise.

**Abbreviations:** AUSCAN, Australian and Canadian Hand Osteoarthritis Index; HADS, Hospital Anxiety and Depression Scale; ITT, intention-to-treat; NRS, Numerical Rating Scale; NSAID, nonsteroidal anti-inflammatory drug.

**Table 3:** Primary and secondary outcomes in ITT population

Outcome at 13 weeks	Pregabalin (N=22)	Duloxetine (N=21)	Placebo (N=22)
NRS			
Baseline (95% CI)	6.1 (5.6 to 6.7)	6.4 (5.7 to 7.1)	6.4 (5.7 to 6.9)
13 weeks (95% CI)	3.4 (2.4 to 4.4)	4.3 (2.6 to 5.9)	5.4 (4.1 to 6.8)
Mean difference (95% CI)	−2.7 (−3.5 to −1.9)	−2.3 (−3.8 to −0.9)	−0.9 (−0.2 to 0.2)
*P*-value	0.023*	0.19	
AUSCAN pain score			
Baseline (95% CI)	317.0 (280.8 to 353.1)	296.0 (248.2 to 343.9)	320.3 (290.9 to 349.6)
13 weeks (95% CI)	176.5 (123.9 to 229.1)	248.1 (162.3 to 333.9)	273.5 (218.0 to 329.0)
Mean difference (95% CI)	−132.1 (−181.1 to −82.9)	−35.8 (−119.7 to 48.2)	−46.61 (−93.9 to 0.75)
*P*-value	0.008*	0.59	
AUSCAN stiffness			
Baseline (95% CI)	60.18 (51.7 to 68.7)	60.95 (46.98 to 74.9)	55.5 (45.2 to 65.8)
13 weeks (95% CI)	36.5 (23.0 to 49.9)	48.25 (29.87 to 66.6)	50.0 (36.0 to 64.0)
Mean difference (95% CI)	−18.7 (−33.1 to −4.3)	−13.5 (−26.5 to −0.6)	−5.67 (−16.8 to 5.5)
*P*-value	0.22	0.96	
AUSCAN function			
Baseline (95% CI)	576.2 (499.1 to 653.4)	577.2 (478.0 to 676.4)	582.3 (509.1 to 655.5)
13 weeks (95% CI)	362.2 (281.7 to 442.7)	496.4 (342.4 to 650.5)	508.7 (379.5 to 637.9)
Mean difference (95% CI)	−246.4 (−341.7 to −151.0)	−101.8 (−248.4 to −44.7)	−67.3 (−156.4 to −21.8)
*P*-value	0.009*	>0.05	
Consumption of rescue medication (total number of days)	9	5	56
HADS			
Anxiety			
Baseline (95% CI)	6.5 (4.6 to 8.4)	5.9 (4.3 to 7.6)	7.2 (5.4 to 9.0)
13 weeks (95% CI)	5.2 (2.9 to 7.5)	4.3 (2.2 to 6.3)	8.2 (6.4 to 9.9)
Mean difference (95% CI)	−0.82 (−2.1 to 0.5)	−1.3 (−3.1 to 0.5)	0.5 (−0.4 to 1.4)
*P*-value	0.15	0.07	
Depression			
Baseline (95% CI)	5.1 (3.6 to 6.7)	4.4 (2.9 to 5.8)	4.9 (3.6 to 6.2)
13 weeks (95% CI)	4.1 (2.6 to 5.6)	3.8 (1.9 to 5.7)	5.1 (3.9 to 6.3)
Mean difference (95% CI)	−1.1 (−2.1 to −0.02)	−0.3 (−1.9 to 1.2)	0.05 (−1.3 to 1.4)
*P*-value	0.66	0.99	

**Notes:**

* Indicates significant at <0.05

**Abbreviations:** AUSCAN, Australian and Canadian Hand Osteoarthritis Index; HADS, Hospital Anxiety and Depression Scale; ITT, intention-to-treat; NRS, Numerical Rating Scale.

**Table 4:** Summary table for per protocol analysis

Outcome at 13 weeks (imputed data on per protocol set)	Pregabalin	Duloxetine	Placebo
NRS			
Baseline (95% CI)	6.1 (5.4 to 6.7)	6.6 (5.7 to 7.4)	6.3 (5.6 to 6.9)
13 weeks (95% CI)	3.4 (2.4 to 4.4)	4.3 (2.6 to 5.9)	5.4 (4.1 to 6.6)
Mean difference (95% CI)	−2.7 (−3.5 to −1.9)	−2.3 (−3.8 to −0.9)	−0.9 (−2.3 to 0.2)
*P*-value	<0.0001*	0.029*	
AUSCAN pain score			
Baseline (95% CI)	308.5 (262.6 to 354.5)	310.6 (254.3 to 367.0)	321.1 (288.7 to 353.4)
13 weeks (95% CI)	176.5 (123.9 to 229.1)	248.1 (162.3 to 333.9)	273.5 (218.0 to 329.0)
Mean difference (95% CI)	−132.0 (−181.1 to −82.9)	−62.5 (−141.6 to 16.6)	−47.1 (−93.8 to 11.7)
*P*-value	0.013*	0.9	
AUSCAN stiffness			
Baseline (95% CI)	59.9 (51.8 to 67.9)	61.8 (45.8 to 77.8)	56.1 (44.5 to 67.7)
13 weeks (95% CI)	36.5 (23.0 to 49.9)	48.3 (29.9 to 66.6)	50.0 (36.0 to 64.0)
Mean difference (95% CI)	−23.4 (−35.7 to −11.1)	−13.5 (−26.5 to −0.6)	5.7 (−16.8 to 5.5)
*P*-value	0.06	0.46	
AUSCAN function			
Baseline (95% CI)	608.5 (541.3 to 675.7)	598.3 (481.2 to 715.3)	580.0 (494.6 to 665.4)
13 weeks (95% CI)	362.2 (281.7 to 442.7)	496.4 (342.4 to 650.5)	508.7 (379.5 to 637.9)
Mean difference (95% CI)	−246.3 (−341.7 to −151.0)	−101.9 (−248.4 to −44.8)	−69.7 (−158.3 to −18.9)
*P*-value	0.02*	0.93	
Consumption of rescue medication (total number of days)	9	5	56
HADS			
Anxiety			
Baseline (95% CI)	6.1 (3.8 to 8.3)	5.6 (3.6 to 7.5)	7.6 (5.8 to 9.5)
13 weeks (95% CI)	5.2 (2.9 to 7.5)	4.3 (2.2 to 6.3)	8.2 (6.4 to 9.9)
Mean difference (95% CI)	−0.82 (−2.1 to 0.5)	−1.3 (−3.1 to 0.5)	0.5 (−0.4 to 1.4)
*P*-value	0.52	0.21	0.71
Depression			
Baseline (95% CI)	5.1 (3.3 to 6.9)	4.1 (2.3 to 5.9)	5.4 (4.0 to 6.7)
13 weeks (95% CI)	4.1 (2.6 to 5.6)	3.8 (1.9 to 5.7)	5.1 (3.9 to 6.3)
Mean difference (95% CI)	−1.1 (−2.1 to −0.02)	−0.3 (−1.8 to 1.2)	0.05 (−1.3 to 1.4)
*P*-value	0.41	0.54	0.93

**Notes:**

* Indicates significant at <0.05

**Abbreviations:** AUSCAN, Australian and Canadian Hand Osteoarthritis Index; HADS, Hospital Anxiety and Depression Scale; NRS, Numerical Rating Scale.

**Table 5:** Side effect profile in all three treatment groups from ITT analysis

System	Pregabalin (N=22)	Duloxetine (N=21)	Placebo (N=22)
Cardiovascular	3	2	1
Digestive	7	18	5
ENT		2	
Endocrine/metabolic	1		
Genitourinary	1		
Hematological			
Mental	9	9	9
Nervous system			
Dry mouth	6	6	4
Headaches	3	8	
Dizziness	7	3	
Sleepiness	5	3	
Loss of balance	7		
Ophthalmological	4	2	1
Respiratory	2	3	
Skin		1	2
Total	55	57	22

**Abbreviations:** ENT, ear, nose and throat; ITT, intention-to-treat.
